# Supplementary material for: Metabolic engineering of oleaginous yeast Rhodotorula toruloides for overproduction of triacetic acid lactone
Source: Biotechnol Bioeng. 2022 Jun 23;119(9):2529–40. doi: 10.1002/bit.28159 (PMC9540541; doi:10.1002/bit.28159)
Supplement: Supplementary file 1 — Supporting information. [file BIT-119-2529-s001.docx]

Supporting Information

**Metabolic Engineering of Oleaginous Yeast *Rhodotorula* *toruloides* for Overproduction of Triacetic Acid Lactone**

Mingfeng Cao^a^, Vinh G. Tran^a^, Jiansong Qin^b^, Andrew Olson^b^, Shekhar Mishra^a^, J. Carl Schultz^a^, Chunshuai Huang^a^, Dongming Xie^b^, and Huimin Zhao^a,c,*^

^a^ Department of Chemical and Biomolecular Engineering, U.S. Department of Energy Center for Bioenergy and Bioproducts Innovation (CABBI), Carl R. Woese Institute for Genomic Biology, University of Illinois at Urbana-Champaign, Urbana, IL 61801, United States

^b^ Department of Chemical Engineering, University of Massachusetts Lowell, Lowell, MA, 01854, USA

^c^ Departments of Chemistry, Biochemistry, and Bioengineering, University of Illinois at Urbana-Champaign, Urbana, IL 61801, United States

*Running title*: Triacetic acid lactone production in *Rhodotorula toruloides*

^*^To whom correspondence should be addressed. Phone: (217) 333-2631. Fax: (217) 333-5052.

E-mail: zhao5@illinois.edu

**Table S1.** List of the main gene targets and DNA sequences of key primers and N20.

| **Targets** | **Protein ID/Notes** | **Primers sequences (5’-3’)**  **(Containing the homologous arms for cloning)** |
| --- | --- | --- |
| ZPK | Gene expression cassette amplification | F-caaattgacgcttagacaac;  R-tatatcctgtcaaacactgatag |
| gRNA | gRNA expression cassette amplification | F-gactatttgcaaagggaaggg;  R-tttttgtgatgctcgtcag |
| *ACL1* | 9726 | F-tacccccatctcccctcctcgtcactcaaccaattgatgtcggcgaaggtgcgtcgaatc;  R-tgggaactactcacacattattatggagaaaactagtttactggcgctgctggacgagga |
| *ACS1* | 14597 | F-atacccccatctcccctcctcgtcactcaaccaattgatggccaaggagcacctctactg;  R-ctgggaactactcacacattattatggagaaaactagttcactcggacgagagcttggct |
| *ALD5* | 12042 | F-tacccccatctcccctcctcgtcactcaaccaattgatgtcggtgcgtcgtgcgcgagtt;  R-tgggaactactcacacattattatggagaaaactagtttagagcgggttcggctgggaga |
| *ACC1* | 8639 | F-atacccccatctcccctcctcgtcactcaaccaattgatgccgtgcgtcgcctccctttc  R-atctgggaactactcacacattattatggagaaaactagtctaggcgaggatgcgggcga |
| *ME1* | 12761 | F-catacccccatctcccctcctcgtcactcaaccaattgatgccctcgaccttcgccccct;  R-aactactcacacattattatggagaaaactagtctactgcgcctgctgctccgcctccaa |
| *PDH-E1* | 13630 | F-tacccccatctcccctcctcgtcactcaaccaattgatgctccgtacgactctcgcaaag;  R-ctgggaactactcacacattattatggagaaaactagtttagacggtcgaggtcatcgac |
| *PDH-E3* | 10040 | F-atacccccatctcccctcctcgtcactcaaccaattgatgctcaaggccaaggacaagtc;  R-ctgggaactactcacacattattatggagaaaactagtttaggcgttgatggctgcgatg |
| *PEX10* | 16017 | F-acccccatctcccctcctcgtcactcaaccaattgatgccagactcgccagcgccagcaa;  R-ctgggaactactcacacattattatggagaaaactagtttacaagttgcgcagaggaagc |
| *PDC1* | 15791 | F-tcatacccccatctcccctcctcgtcactcaaccaattgatgccagtttcggtcaccgtg;  R-ctgggaactactcacacattattatggagaaaactagttcacgccgcattcaactcggca |
| *AMPD1* | 11469 | F-catacccccatctcccctcctcgtcactcaaccaattgatggctgacacaatagattcac;  R-atctgggaactactcacacattattatggagaaaactagtctacctcccattcacgctgg |
| **Targets** | **Protein ID** | **N20 sequences for SgRNA design** |
| *LRO1* | 16477 | gagttcggtgacgttgagag |
| *DGA1* | 16460 | ttgcgccctttggcgtcccg |
| *CIT2* | 11331 | gctcggggatgatctccttg |
| *MLS1* | 9457 | cgggggtgaggatctcttgg |
| *NTE1* | 14309 | cgtcgcgagctatgtctgga |
| *YIA6* | 10961 | tcaagggcgccgtgtggttt |

**Table S2.** DNA sequences of the genes expressed in *R. torulodies*.

| **Genes** | **Sequences** |
| --- | --- |
| *GhPS* | atgggctcgtactcgtcggacgacgtcgaggtcatccgcgaggctggccgcgctcagggcctcgccaccatcctcgccatcggcaccgccacgccgccgaactgcgtcgcccaggccgactacgcggactactacttccgcgtcaccaagtcggagcacatggtcgacctcaaggagaagttcaagcgcatctgcgagaagaccgccatcaagaagcgctacctcgcgctcaccgaggactacctccaggagaaccccaccatgtgcgagttcatggccccgtcgctcaacgcccgccaggacctcgtcgtcacgggcgtcccgatgctcggcaaggaggccgccgtcaaggccatcgacgagtggggcctcccgaagtcgaagatcacccacctcatcttctgcaccaccgctggcgtcgacatgcctggcgccgactaccagctcgtcaagctcctcggcctctcgccgtcggtcaagcgctacatgctctaccagcagggctgcgccgctggcggcaccgtcctccgcctcgctaaggacctcgccgagaacaacaagggctcgcgcgtcctcatcgtctgctcggagatcaccgccatcctcttccacggcccgaacgagaaccacctcgactcgctcgtcgcgcaggccctcttcggcgacggcgctgcggcgctcatcgtcggctcgggccctcacctcgccgtcgagcgcccgatcttcgagatcgtctcgaccgaccagaccatcctcccggacaccgagaaggccatgaagctccacctccgcgagggcggcctcaccttccagctccaccgcgacgtcccgctcatggtcgccaagaacatcgagaacgccgcggagaaggcgctctcgcccctcggcatcaccgactggaactcggtcttctggatggtccaccctggcggccgcgcgatcctcgaccaggtcgagcgcaagctcaacctcaaggaggacaagctccgcgcctcgcgccacgtcctctcggagtacggcaacctcatctcggcctgcgtcctcttcatcatcgacgaggtccgcaagcgctcgatggccgagggcaagtcgaccaccggcgagggcctcgactgcggcgtcctcttcggcttcggccctggcatgaccgtcgagaccgtcgtcctccgctcggtccgcgtcacggccgctgtcgccaacggcaactag |
| *VvPS* | atgggctcgggcacggtcgagcaggtcggctcgctcaagaaggcgaacccgggcaaggcgacgatcctcgcgctcggcaaggcgttcccgcaccagctcgtcatgcaggagttcctcgtcgacggctacttccgcaacacgaactgcgacgacccggacctcaaggagaagctcgcgcgcctctgcaagacgacgacggtcaagacgcgctacgtcgtcatgtcggaggagatcctccgcaagtacccggagctcgtcatcgagggccagccgacggtcaagcagcgcctcgacatctgcaacaaggcggtcacgcagatggcgatcgacgcgtcgaaggcgtgcatcaagaactggggccgctcggtctcggagatcacgcacctcgtctacgtctcgtctagtgaagctcgacttcctggaggagacttgtacttggcgaagggacttggcctctcgccggagacgcaccgcgtccagctctacttcatgggttgctctggaggtgttgctggtcttcgtgttgcaaaggacatcgctgaaaacaacccggagtcgcgcgtcctcctcgcgacgtcggagacgacgatcatcggcttcaagccaccttctgctgacaggccatacgaccttgttggagttgctcttttcggagacggtgctggagcaatgatcatcggctcggacccgatcccgtcgacggagcgcccgctcttcgagctccacacggcgatccagaacttcctcccggacacggagaagacgatcgacggccgcctcacggaggagggcatctcgttcaagctcgcgcgcgagctcccgcagatcatcgaggaccacatcgaggcgttctgcgacaagctcatccgcaacgtcggcttctcggacgaggactacaacaagatcttctgggcggtccacccgggcggcccggcgatcctcaaccgcatggagaagcgcctcgacctcctcccggagaagctcaacgcgtcgcgccgcgcgctcgcggactacggcaacgcgtcgtcgaacacgatcgtctacgtcctcgagtacatgctcgaggagtcgtcgaagacgaagcgccaggaccagggcgacggcgagtggggcctcatcctcgcgttcggcccgggcatcacgttcgagggcatcctcgcgcgcaacctcacggtctag |
| *SmPS* | atgtcgcacgagacggcgtcgacgtacatcccgtcgacgggcctcccggcgccggtctcgccgaaggtctacatcacgggcctcgcggcgcagtacccgccgtacctcttccgcccgtcggacctcgacacgctcgcggcgcagctccacgacccgtcgaacccgggcatccagcgcctcctccacctcaaccgcaagacgggcatcctcacgctcccgtcggtcctcccgtcgacgtcgttcccgtcgccgtcgccgccgacgatcacggagctcgacctcacgttccgccaccacggaacagctcttgctgttcaagcttgccactcagcattggctgagtcgcaccacctcccgtcggacatcacgcacacggtcgcggtcacgtgcacgtcgacgggctgcccgggcttcgacctcctcgttgctcagactcttggtcttgcacctacagtagaccgaactttgttgcacggagttggatgcgcaggaggtttggctattcttcgagttgctgcgcagatcgttgcaggtgcaactgctcgaggaggtgctgctgttgttttggcatacgcgtgcgaactttgcacacctttggttcgacatgctttggttgaggctgcaaggagggagccaggacaagttggaatcgaaggtgctcttttctcagacggagctgccggtgtcgttgtctgcaacgcgatgggcaagggtggtagggaagctgttttcgaggttggagcttggggtactgaaactgtaccaggaactgttggagaaatgggcttcttcacggagggcttcggctaccagacgacgctcacaaggaacgttcctttgattgcacgaggtgctatgagaggaatgttcgagcgcctcgtcggcgagtacaagtcggagttcggcgaggaggtcggcggctctgaggattttgactgggcattgcaccctggtggagctgcaattatggacggtgttcgagacgtaatgggcctctcggagcaccagctccgcgcgtcgcgcgaggtctaccgcatcaagggcaactcgtcgtcgccgacggtcctcatcgtcctcgacaagctccgccgcatgtcgccgggcaaggagcacgtcatcgcgacgtcgttcggcccgggcgtcacgatcgagatggtcgtcctccgcaagtcgtcgggcacgtgctag |
| *AoPS* | atgatcgagccgctcccgacggaggacatcccgaagcagtcggtctcgatcgttggtatcgctagtcgatgcgctcctcacaagttgggtgctgacgagttggaggcgatcgcgcgccgccactactcatctaccccttcgcttgaaaagatgctcgagatcaaccgcaagacgcgcatcgaccaccgctactcggtcttctcgtcggaccacgagcactggcaccgcccgacgatcccgtcgttctcggagtgcgactcgctcttcaaggagtacggaatccctcttgcttcagcagcttctgctagggcaattcaggactggggtggagttcctgacgaaatcacgcacctcgtcgcggtcacgtgcacgaacacggcgcacccgggcttcgactcggtcctctgccgcaagctcggcctcaagtgcaacgttcgaagggttttgcttcacggaatcggttgcggaggaggaatctcagcaatgcgtgttgctcacgaattgcttcttggttctactcagcagggagttcctgctcgtgcattgatcgttgcttgcgaggttcctactgttttcgcgcgctcggagctcgacatcatggacaagacgcaggacgtcaacgtcgcgatgtgcctcttcggcgactgcgcggcggcgctcgtcctctcgaacggcatcggccacaaggcgtcggagcagcgcccgatctggaacatcctcaactgcgagccgacgcagttcgacggcacggaggacatcgcgcacttcaacgtccacgacaagggctaccacgcgatcatcgacaagcgcatcccgcagctcacgggcaagtgcgtcccggcgggcttccagtcgctcatctcgagcactccctcgctggcgctcgaggagaagaactacgtcccgtcgaactatggatgggcagttcatccaggaggttacgctgttcttgttgcagctcaagacgcgcttggacttactgcagacgaccttcgagcatcgtacgacgcgtaccgcgacggcggcaacacgatctcgacgacgatcatccgcatcctcgagaagctccgcgacgagcacaagcacggctcgaaccagaaggacaagctcgtcctcgcggcgatcggccacggcatcacgctcgagacggcgatcctcacgcgcccgggctcgtcgtcgtacctccacgcgtag |
| *YLACL1* | atgtcggccaacgagaacatctcgcgcttcgacgccccggtcggcaaggagcacccggcctacgagctcttccacaaccacacccgctcgttcgtctacggcctccagccgcgcgcctgccagggcatgctcgacttcgacttcatctgcaagcgcgagaacccgtcggtcgccggcgtcatctacccgttcggcggccagttcgtcaccaagatgtactggggcaccaaggagaccctcctcccggtctaccagcaggtcgagaaggccgccgccaagcacccggaggtcgacgtcgtcgtcaacttcgcctcgtcgcgctcggtctactcgtcgaccatggagctcctcgagtacccgcagttccgcaccatcgccatcatcgccgagggcgtcccggagcgccgcgcccgcgagatcctccacaaggcccagaagaagggcgtcaccatcatcggcccggccaccgtcggcggcatcaagccgggctgcttcaaggtcggcaacaccggcggcatgatggacaacatcgtcgcctcgaagctctaccgcccgggctcggtcgcctacgtctcgaagtcgggcggcatgtcgaacgagctcaacaacatcatctcgcacaccaccgacggcgtctacgagggcatcgccatcggcggcgaccgctacccgggcaccaccttcatcgaccacatcctccgctacgaggccgacccgaagtgcaagatcatcgtcctcctcggcgaggtcggcggcgtcgaggagtaccgcgtcatcgaggccgtcaagaacggccagatcaagaagccgatcgtcgcctgggccatcggcacctgcgcctcgatgttcaagaccgaggtccagttcggccacgccggctcgatggccaactcggacctcgagaccgccaaggccaagaacgccgccatgaagtcggccggcttctacgtcccggacaccttcgaggacatgccggaggtcctcgccgagctctacgagaagatggtcgccaagggcgagctctcgcgcatctcggagccggaggtcccgaagatcccgatcgactactcgtgggcccaggagctcggcctcatccgcaagccggccgccttcatctcgaccatctcggacgaccgcggccaggagctcctctacgccggcatgccgatctcggaggtcttcaaggaggacatcggcatcggcggcgtcatgtcgctcctctggttccgccgccgcctcccggactacgcctcgaagttcctcgagatggtcctcatgctcaccgccgaccacggcccggccgtctcgggcgccatgaacaccatcatcaccacccgcgccggcaaggacctcatctcgtcgctcgtcgccggcctcctcaccatcggcacccgcttcggcggcgccctcgacggcgccgccaccgagttcaccaccgcctacgacaagggcctctcgccgcgccagttcgtcgacaccatgcgcaagcagaacaagctcatcccgggcatcggccaccgcgtcaagtcgcgcaacaacccggacttccgcgtcgagctcgtcaaggacttcgtcaagaagaacttcccgtcgacccagctcctcgactacgccctcgccgtcgaggaggtcaccacctcgaagaaggacaacctcatcctcaacgtcgacggcgccatcgccgtctcgttcgtcgacctcatgcgctcgtgcggcgccttcaccgtcgaggagaccgaggactacctcaagaacggcgtcctcaacggcctcttcgtcctcggccgctcgatcggcctcatcgcccaccacctcgaccagaagcgcctcaagaccggcctctaccgccacccgtgggacgacatcacctacctcgtcggccaggaggccatccagaagaagcgcgtcgagatctcggccggcgacgtctcgaaggccaagacccgctcgtag |
| *NAT^R^* | atggcggccgccactcttgacgacacggcttaccggtaccgcaccagtgtcccgggggacgccgaggccatcgaggcactggatgggtccttcaccaccgacaccgtcttccgcgtcaccgccaccggggacggcttcaccctgcgggaggtgccggtggacccgcccctgaccaaggtgttccccgacgacgaatcggacgacgaatcggacgccggggaggacggcgacccggactcccggacgttcgtcgcgtacggggacgacggcgacctggcgggcttcgtggtcgtctcgtactccggctggaaccgccggctgaccgtcgaggacatcgaggtcgccccggagcaccgggggcacggggtcgggcgcgcgttgatggggctcgcgacggagttcgcccgcgagcggggcgccgggcacctctggctggaggtcaccaacgttaacgcaccggctatccacgcgtaccggcggatggggttcaccctctgcggcctggacaccgccctgtacgacggcaccgcctcggacggcgagcaggcgctctacatgagcatgccctgcccctaa |
| *Hyg^R^* | atgaagaagccggagctcaccgccacctcggtcgagaagttcctcatcgagaagttcgactcggtgtcggacctcatgcagctctcggagggcgaggagtcgcgcgccttctcgttcgacgtcggcggccgcggctacgtcctccgcgtcaactcgtgcgccgacggcttctacaaggaccgctacgtctaccgccacttcgcctcggccgccctcccgatcccggaggtcctcgacatcggcgagttctcggagtcgctcacctactgcatctcgcgccgcgcccagggcgtcaccctccaggacctcccggagacggagctcccggccgtcctccagccggtcgccgaggcgatggacgccatcgccgccgccgacctctcgcagacctcgggcttcggcccgttcggcccgcagggcatcggccagtacaccacctggcgcgacttcatctgcgccatcgccgacccgcacgtctaccactggcagaccgtcatggacgacaccgtctcggcctcggtcgcccaggccctcgacgagctcatgctctgggccgaggactgcccggaggtccgccacctcgtccacgccgacttcggctcgaacaacgtcctcaccgacaacggccgcatcaccgccgtcatcgactggtcggaggccatgttcggcgactcgcagtacgaggtcgccaacatcttcttctggcgcccgtggctcgcctgcatggagcagcagacccgctacttcgagcgccgccacccggagctcgccggctcgccgcgcctccgcgcctacatgctccgcatcggcctcgaccagctctaccagtcgctcgtcgacggcaacttcgacgacgccgcctgggcgcagggccgctgcgacgccatcgtccgctcgggcgccggcaccgtcggccgcacccagatcgcccgccgctcggccgccgtctggaccgacggctgcgtcgaggtcctcgccgactcgggcaaccgccgcccgtcgacccgcccgcgcgccaaggagtag |
| *G418^R^* | atgggcaaggagaagacgcacgtctcgcgcccgcgcctcaactcgaacatggacgccgacctctacggctacaagtggtcccgcgacaacgtcggccagtcgggcgccacgatctaccgcctctacggcaagccggacgccccggagctcttcctcaagcacggcaagggctcggtcgccaacgacgtcacggacgagatggtccgcctcaactggctcacggagttcatgccgctcccgacgatcaagcacttcatccgcacgccggacgacgcctggctcctcacgacggccatcccgggcaagacggccttccaggtcctcgaggagtacccggactcgggcgagaacatcgtcgacgccctcgccgtcttcctccgccgcctccactcgatcccggtctgcaactgcccgttcaactcggaccgcgtcttccgcctcgcccaggcccagtcgcgcatgaacaacggcctcgtcgacgcctcggacttcgacgacgagcgcaacggctggccggtcgagcaggtctggaaggagatgcacaagctcctcccgttctcgccggactcggtcgtcacgcacggcgacttctcgctcgacaacctcatcttcgacgagggcaagctcatcggctgcatcgacgtcggccgcgtcggcatcgccgaccgctaccaggacctcgccatcctctggaactgcctcggcgagttctcgccgtcgctccagaagcgcctcttccagaagtacggcatcgacaacccggacatgaacaagctccagttccacctcatgctcgacgagttcttctag |

**Table S3.** Substrate utilization test.

| **Media** | **Cell OD** | **Residual substrates** | **TAL titer (g/L)** |
| --- | --- | --- | --- |
| YPD | 68±3.2 | 0 | 1.97±0.12 |
| YPG | 35±1.5 | 9.7±0.5 | 1.17±0.06 |
| YPX | 71±1.5 | 0 | 2.13±0.15 |
| YPS | 45±1.2 | 12.4±0.8 | 1.07±0.06 |
| SC | 19±1.0 | 10.5±0.7 | 0.47±0.06 |
| YPDX | 62±2.6 | 0 | 2.23±0.06 |
| YP2D | 83±2.1 | 0 | 3.63±0.15 |
| YPD+0.5%NaAc | 68±3.5 | 0.4±0.1 | 3.33±0.12 |
| YPD+1%NaAc | 62±2.1 | 4.5±0.8 | 3.57±0.12 |
| YPD+2%NaAc | 54±2.6 | 13.6±0.4 | 3.6±0.26 |
| YP-NaAc | 23±2.6 | 15.6±0.6 | 0.84±0.09 |

**Table S4.** TAL production of various metabolically engineered *R. toruloides* strains.

| **Overexpressed targets** | **Titer (g/L)** | **Deleted targets** | **Titer**  **(g/L)** | **Combined targets** | **Titer**  **(g/L)** |
| --- | --- | --- | --- | --- | --- |
| I12 | 4.56±0.26 | I12-*Cas9* | 4.70±0.20 | I12-*ACL1* | 5.37±0.13 |
| *ACC1* | 4.84±0.14 | *CIT2∆* | 5.20±0.18 | *ACL1-PEX10* | 5.83±0.42 |
| *2GhPS* | 5.06±0.11 | *MLS1∆* | 5.47±0.21 | *ACL1-PDC2* | 5.59±0.30 |
| *PDH-E1* | 5.27±0.18 | *LRO1∆* | 5.42±0.11 | *ACL1-ACS1* | 5.81±0.40 |
| *PDH-E3* | 5.30±0.38 | *DGA1∆* | 5.05±0.26 | *ACL1-PDH-E1* | 5.80±0.31 |
| *PDC1* | 5.13±0.26 | *PYC1∆* | 4.42±0.34 | *ACL1-PDH-E3* | 5.97±0.22 |
| *ALD5* | 5.51±0.20 | *NTE1∆* | 4.81±0.32 | *ACL1-ACC1* | 6.94±0.42 |
| *ACS1* | 4.59±0.47 | *YIA6∆* | 4.82±0.25 | *ACL1-ALD5* | 6.33±0.27 |
| *ACL1* | 6.61±0.30 |  |  | *ACL1-ACL1* | 5.98±0.32 |
| *AMPD1* | 3.83±0.56 |  |  |  |  |
| *ME1* | 4.60±0.22 |  |  |  |  |
| *PEX10* | 5.10±0.46 |  |  |  |  |
| *YLACL1* | 5.06±0.27 |  |  |  |  |

**Table S5.** Fed-batch bioreactor fermentation of *R. toruloides* I12-*ACL1*-*ACC1* using glucose or oilcane juice.

| **Time (h)** | **Cell OD_600_** | **Glucose consumed (g/L)** | **Acetate consumed (g/L)^*^** | **TAL titer (g/L)** | **Yield (g/g)** | **Yield (%)^**^** |
| --- | --- | --- | --- | --- | --- | --- |
| 0.0 | 0.5 | 0 | 0 | 0 | 0 | 0 |
| 23.0 | 43.2 | 48.9 | 0 | 2.40 | 0.049 | 10.53 |
| 36.0 | 86.5 | 106.3 | 0 | 3.91 | 0.037 | 7.89 |
| 47.0 | 121.6 | 144.5 | 3.25 | 5.15 | 0.035 | 7.48 |
| 71.0 | 132.4 | 195.6 | 28.9 | 8.98 | 0.040 | 8.58 |
| 95.0 | 144.5 | 217.7 | 83.1 | 22.1 | 0.073 | 15.76 |
| 118.0 | 135.2 | 223.8 | 126.9 | 27.7 | 0.079 | 16.97 |
| 130.5 | 145.0 | 232.8 | 145.6 | 28.0 | 0.074 | 15.88 |
| 143.0 | 138.0 | 238.9 | 165.0 | 24.0 | 0.059 | 12.73 |
| **Time (h)** | **Cell OD_600_** | **Sugars consumed (g/L)** | **Acetate consumed (g/L)** | **TAL titer (g/L)** | **Yield (g/g)** | **Yield (%)*** |
| 0 | 0.2 | 0 | 0.00 | 0.00 | 0 | 0 |
| 24 | 58.7 | 52.7 | 0.41 | 2.75 | 0.052 | 11.1 |
| 48 | 84.0 | 100.4 | 19.3 | 7.98 | 0.067 | 14.3 |
| 71.5 | 125.0 | 131.0 | 35.6 | 13.3 | 0.080 | 17.1 |
| 96 | 128.2 | 163.4 | 49.6 | 17.2 | 0.081 | 17.3 |
| 120.5 | 122.0 | 194.4 | 61.4 | 22.9 | 0.089 | 19.2 |
| 144 | 114.8 | 225.5 | 67.9 | 20.4 | 0.069 | 14.9 |

Note: ^*^The consumed acetate included both NaAc and acetic acid (pH adjustment); ^**^The theoretical maximum yield of TAL produced from glucose/acetate was 0.47 g/g.

**Fig. S1.** Cell growth assay of *R. toruloides* in YPD media supplemented with varying TAL concentrations (0, 3, 5, 7, and 9 g/L). This assay was carried out using a Bioscreen C (Growth Curves USA, Piscataway, NJ). The wild-type strain IFO0880 was inoculated at OD_600_ of 0.2 into 200 µL of YPD media with 0, 3, 5, 7, and 9 g/L of TAL concentration. Cells were grown with continuous shaking for 80 hours at 30 °C. OD_600_ was measured every hour, and the experiment was performed in biological triplicate.

**Fig. S2.** The UV-Vis absorbance (**A**) and LC-MS (**B**) profile of TAL produced by *R. toruloides* I12 strain.

**Fig. S3.** TAL production by the 2^nd^ round genome integration of *GhPS*. To test the variation among colonies, 10 different colonies were picked and cultured in 3 mL of YPD for 72 h in culture tube.

**Fig. S4.** TAL production by I12-*ACL1-ACC1* and I12-*ACL1* in shake flask.

**Fig. S5.** Production of TAL and intracellular lipids by I12-*ACL1-ACC1* in shake flask at 96 h using YP2D and YP2D+0.5% NaAc. (**A**) The production of intracellular lipids (mg/ g DCW); (**B**) Production comparison of TAL and the main lipids components (TAG).

**Materials and methods**

**Lipidomic analysis via liquid chromatography – mass spectrometry**

At the time of sampling, cell concentration of the culture was measured as OD_600_/mL. One mL of cell culture was harvested, washed once with 150 mM ammonium bicarbonate (ABC) buffer (pH = 8), then resuspended in 500 µL ABC, and lysed open with approximately 200 µL of 0.5 µm zirconium glass beads via high-speed vortexing for 30 minutes. For lipid extraction, 2 OD_600_ units of cell lysate were added to a glass tube containing 200 µL ABC, 1 mL of 2:1 chloroform:methanol and 12 µL of an internal standard cocktail. The glass tube was vortexed in a Fisherbrand MultiTube Vortexer (Thermo Fisher Scientific, Waltham, MA) for 2 hours at 2500 rpm. After the phases were clearly separated, the chloroform layer was separated into a fresh vial and dried overnight. The dried lipid extract was resuspended in 100 µL of 4:2:1 isopropanol:methanol:chloroform. 10 µL of the lipid extract was injected on an LC-MS instrument (Vanquish UHPLC and Q-Exactive Orbitrap, Thermo Fisher Scientific, Waltham, MA).

The LC-MS protocol was followed as described in (Zhang et al., 2018). LC separation was performed on a Thermo Accucore Vanquish C18+ column (2.1 × 150 mm, 1.5 μm) with mobile phase A (60% acetonitrile: 40% H_2_O with 10 mM ammonium formate and 0.1% formic acid) and mobile phase B (90% isopropanol: 10% acetonitrile with 10 mM ammonium formate and 0.1% formic acid) and a flow rate of 0.25 mL/min. The linear gradient was as follows: 0 min, 60% A; 12 – 13.5 min, 0% A; 14 – 16 min, 60% A. The gas flow rates and MS1/MS2 scan parameters were followed exactly as listed in (Zhang et al., 2018). Data processing of the .RAW files generated from LC-MS runs was performed using the mzMine software (Pluskal et al., 2010). Identity of lipids was ascertained by comparing spectra to an in-house database of lipid molecules. Finally, quantification was performed using a one-point calibration where the peak intensity of each lipid molecule was normalized to the intensity of the representative lipid for its class within the spiked-in internal standard cocktail. This normalized value was multiplied to the absolute mole amount of the internal standard.

Separately, a calibration curve of OD_600_ units versus grams of dry cell weight (g DCW) was constructed for each culture condition. This calibration curve was used to convert absolute lipid amounts from pmol/OD_600_-units to mg/g DCW.

**References**

Pluskal, T., Castillo, S., Villar-Briones, A., & Oresic, M. (2010). MZmine 2: modular framework for processing, visualizing, and analyzing mass spectrometry-based molecular profile data. *BMC Bioinformatics*, *11*, 395. <https://doi.org/10.1186/1471-2105-11-395>

Zhang, H., Freitas, D., Kim, H. S., Fabijanic, K., Li, Z., Chen, H., Mark, M. T., Molina, H., Martin, A. B., Bojmar, L., Fang, J., Rampersaud, S., Hoshino, A., Matei, I., Kenific, C. M., Nakajima, M., Mutvei, A. P., Sansone, P., Buehring, W., Wang, H., Jimenez, J. P., Cohen-Gould, L., Paknejad, N., Brendel, M., Manova-Todorova, K., Magalhães, A., Ferreira, J. A., Osório, H., Silva, A. M., Massey, A., Cubillos-Ruiz, J. R., Galletti, G., Giannakakou, P., Cuervo, A. M., Blenis, J., Schwartz, R., Brady, M. S., Peinado, H., Bromberg, J., Matsui, H., Reis, C. A., & Lyden, D. (2018). Identification of distinct nanoparticles and subsets of extracellular vesicles by asymmetric flow field-flow fractionation. *Nat Cell Biol*, *20*(3), 332-343. <https://doi.org/10.1038/s41556-018-0040-4>
